# COmbination of Targeted temperature management and Thrombectomy after acute Ischemic Stroke (COTTIS) to improve favorable neurological outcome: a matched-pair analysis

**DOI:** 10.1093/esj/aakaf031

**Published:** 2026-02-17

**Authors:** Wolf-Dirk Niesen, Linda Stephan, Johann Lambeck, Mohammad Fazel, Christian Taschner, Jürgen Bardutzky

**Affiliations:** Department of Neurology and Neuroscience, Medical Center, University of Freiburg, Faculty of Medicine, University of Freiburg, Freiburg, Germany; Department of Neurology and Neuroscience, Medical Center, University of Freiburg, Faculty of Medicine, University of Freiburg, Freiburg, Germany; Department of Neurology and Neuroscience, Medical Center, University of Freiburg, Faculty of Medicine, University of Freiburg, Freiburg, Germany; BrainCool AB, Clinical Studies, Lund, Sweden; Department of Neuroradiology, Medical Center, University of Freiburg, Faculty of Medicine, University of Freiburg, Freiburg, Germany; Department of Neurology and Neuroscience, Medical Center, University of Freiburg, Faculty of Medicine, University of Freiburg, Freiburg, Germany

**Keywords:** Stroke, hypothermia, mechanical thrombectomy, large vessel occlusion

## Abstract

**Introduction:**

Despite the considerable success of endovascular treatment (EVT) in stroke due to large vessel occlusion (LVO), many patients still experience poor outcomes, highlighting the need for additional therapeutic approaches. In a preliminary study [COmbination of Targeted temperature management and Thrombectomy after acute Ischemic Stroke (COTTIS trial)] safety and feasibility of rapid transnasal cooling to 35°C prior to EVT in LVO was established. The objective of this investigation was to determine whether this combination improves outcomes based on a matched-pair analysis of our prospective EVT registry.

**Patients and methods:**

A single-center matched-pair analysis was conducted on patients with EVT in LVO. Hypothermic patients were recruited from the prospective COTTIS trial. Normothermic patients were recruited from a prospective stroke registry on EVT in LVO at our institution. Matching parameters were age, gender, National Institutes of Health Stroke Scale (NIHSS), Alberta Stroke Program Early CT Score (ASPECTS), occlusion type/side and Thrombolysis in Cerebral Infarction (TICI) score. Primary outcome was a favorable neurological outcome [modified Rankin Score (mRS) of 0–2] at 90 days.

**Results:**

A total of 66 patients were analyzed (hypothermia: 22; standard care: 44). Temperature profiles differed by a mean of 1°C. A favorable neurological outcome was reached more often in hypothermia (68.2%) compared with standard care (29.5%) [odds ratio 5.1 (95% confidence interval 1.69; 15.38)] (*p* = 0.004). In the shift analysis in hypothermic patients the probability of attaining a higher score on the mRS was reduced significantly. Safety outcomes did not differ between groups.

**Conclusion:**

Our comparative analysis on periinterventional mild hypothermia in EVT suggests a beneficial effect on clinical outcome. Despite the small sample size the effect size is surprisingly high, which needs critical consideration. Randomized controlled trials are needed to validate these findings.

**Trial Registration:**

The COTTIS study was registered at DKRS: DRKS-ID DRKS00023573.

## Graphical Abstract

**Graphical Abstract:**
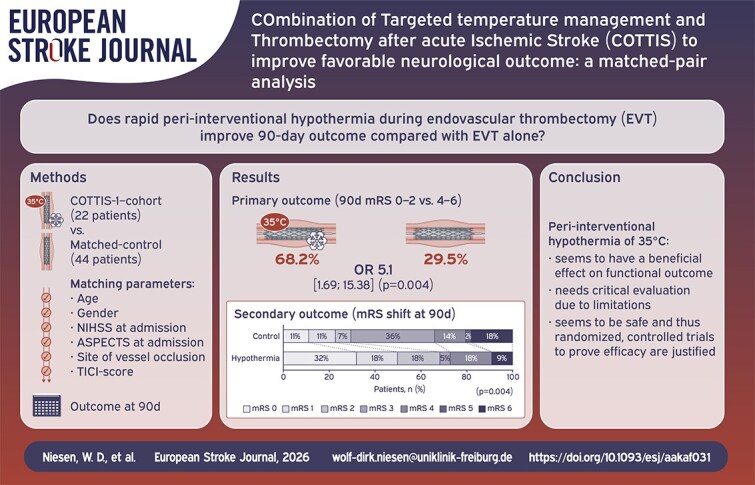

## Introduction

Introducing endovascular treatment (EVT) in acute stroke treatment of large anterior vessel occlusion (LVO) stroke has significantly improved outcome in this patient group and has been proven to be beneficial in acute in LVO up to 24 h from symptom onset.^1–3^ However, despite recanalization rates of >80%, treatment outcomes remain limited; more than half of the patients undergoing EVT remain functionally dependent and one-third of patients are bedridden or dead within 90 days of stroke onset.^1,4^ In these patients, the extent of late infarct growth and final infarct volume have been identified as predictors for clinical outcome after successful recanalization therapy and depend on quality of collateral flow^5^ as well as on time from onset to recanalization.^6^ Furthermore, reperfusion itself has been shown to trigger a series of pathological processes, including astrocyte swelling, pericyte contraction and platelet aggregation, ultimately leading to microcirculatory failure, known as the ‘no-reflow’ phenomenon.^7,8^ Consequently novel treatment approaches must therefore aim to reduce secondary infarct growth, increase penumbral salvage and enhance the effectiveness of reperfusion therapies. The impact of temperature on infarct growth is a well-known phenomenon with a negative influence of temperatures exceeding 37.5°C.^9^ Data from animal studies proved a high degree of efficacy. The administration of mild hypothermia during the ultra-early phase of acute stroke resulted in a reduction of infarct growth by up to 50%.^10^ In short, interaction between energy supply and energy demand determines infarct progression and its growth velocity in the hypoperfused tissue. Hypothermia reduces cerebral metabolism and oxygen consumption. Thus, by reducing cerebral oxygen demand, hypothermia might be the neuroprotectant to shift this balance towards energy supply.^11^ On a cellular level, hypothermia targets a multitude of ischemia-induced pathways, which include energy depletion, ion shifts, inflammation and free radical formation.^12^ By reducing cerebral oxygen consumption, hypothermia can lead to preservation of penumbral tissue. This effect is already seen with small changes of temperature reductions.^13^ In summary, the strongest correlations for the beneficial effects of hypothermia were described when it was administered in the intra-ischemic phase, with temporary occlusion^10^ and already with small decreases in temperature.^14^ The clinical implementation of hypothermia for acute ischemic stroke (AIS) proved unsuccessful for a number of reasons. These include prolonged time to target temperature, side effects, focus on anti-edema treatment instead of neuroprotection and clinical trials with small sample size. There are only few data pointing out that patients who undergo mild hypothermia (34.5°C) after successful endovascular treatment might have less cerebral edema and hemorrhagic transformation, and better outcomes.^15^ Inducing mild hypothermia prior to EVT in AIS imitates preclinical studies by using the neuroprotective effects of hypothermia before and after recanalization, thereby additionally targeting reperfusion injury following successful recanalization.^16^ Recently, the COmbination of Targeted temperature management and Thrombectomy after acute Ischemic Stroke (COTTIS) trial demonstrated that hypothermia to the target temperature of 35°C is feasible and was reached without time delay using a novel transnasal cooling technique. Additionally, neurological outcome in this cohort seemed to be superior to those in large randomized trials and previous smaller trials at the same institution.^17^

To test the hypothesis that prompt initiation of mild hypothermia combined with revascularization therapy (EVT) helps to further improve neurological outcome after large-vessel stroke, we conducted a secondary match-pair-analysis in patients with LVO in the anterior circulation undergoing EVT and compared functional outcome in the hypothermia vs normothermia group.

## Patients and methods

### Study design

A secondary matched-pair analysis was conducted on stroke patients with LVO in the anterior circulation and indication for EVT by comparing 90-day outcomes of patients from (i) a prospective, single-arm, open-label clinical trial testing the feasibility and safety of periinterventional hypothermia in patients with LVO^17^ (interventional group) and (ii) a set of matched patients with LVO and EVT who had not received periinterventional hypothermia (control group). Patients in the control group were derived from our prospective single stroke center database on interventionally treated stroke patients between 2012 and 2022. The neurological outcome was compared using the modified Rankin scale (mRS) at 90 days.

### Patients

#### Interventional group

All patients in the interventional group participated in the COTTIS trial (for details of trial design, protocol and inclusion and exclusion criteria see Bardutzky *et al*.^17^). In summary, the interventional group comprised acute stroke patients with a National Institutes of Health Stroke Scale (NIHSS) of > 5 due to LVO who underwent EVT within 24 h of symptom onset in accordance with the current guidelines of the European Stroke Organization.^18^ EVT technique was performed according to current guidelines and selected at the discretion of the interventionalist on an individual patient basis. All patients in the interventional group were intubated before interventional stroke treatment and received immediate transnasal cooling following intubation using the RhinoChill nasal catheter and system with a target temperature of 35°C. Subsequent to recanalization body temperature was maintained at 35°C for 6 h followed by a period of controlled rewarming at a rate of 0.2°C/h utilizing surface cooling (Brain-Cool). To prevent shivering patients were intubated and sedated for the whole cooling period according to a standard protocol. When patients reached a body temperature of 36°C, analgosedation was discontinued and patients were extubated as soon as possible. When body temperature reached 36.5°C controlled rewarming was terminated.

To monitor the temperature profile during hypothermia and rewarming, temperature was monitored continuously by an esophageal temperature probe (Mon-a-Therm, Covidien, MA, USA) as well as discontinuously by tympanic temperature measurement every 15 min during EVT and at defined time points during intensive care unit (ICU) stay.

#### Control group

Patients from the control group were extracted from a prospective single stroke center stroke registry at the same institution as the interventional group using individual matched sampling. Inclusion criteria were consistent for both groups (see Bardutzky *et al*.^17^), e.g., age >18 years, pre-stroke mRS of 0–2, symptom onset within 24 h , NIHSS >5, intracranial occlusion of the M1 or M2 segment of the middle cerebral artery or internal carotid artery or tandem occlusion on computed tomography/magnetic resonance angiography. This stroke registry contains 1116 consecutive patients who underwent EVT for LVO from January 2012 to March 2022. Patients in the control group underwent standard EVT and standard stroke treatment according to our institutional protocol, which includes EVT in general anesthesia and intubated admission to our neurointensive care unit. No interferences in temperature were carried out except for temperature-targeted management in case of fever. The data were finalized through a subsequent review of the institutional electronic patient charts and documentation systems.

Given the absence of a predefined temperature monitoring protocol within the control group, all measured temperatures were identified through a comprehensive review of the patients’ electronic medical records. Temperature measurements were recorded using tympanic thermometers. Temperatures were included up to 12 h after admission and then categorized into a series of defined time points: T0 (patient temperature at admission), T1 (3 h after admission), T2 (6 h after admission) and T3 (12 h after admission).

#### Matching parameters

In order to ensure an equitable distribution of potential cofounders, a set of matching factors were defined. These factors included age, gender, NIHSS at admission, Alberta Stroke Program Early CT Score (ASPECTS) at admission, site of vessel occlusion and Thrombolysis in Cerebral Infarction (TICI) score. Patients were included if they met the defined inclusion criteria of COTTIS. To increase the power of the study, we matched two controls for every case patient in the interventional group (2:1 matching). The following ranges were defined for the matching factors using nearest neighbor matching:

- maximum deviation range for ASPECTS ±1
- maximum deviation range for NIHSS ±3 points
- maximum deviation range for age 5 years
- maximum deviation range for TICI dichotomized vs one grade

Patients were excluded if data were missing or matching criteria deviated beyond the set ranges.

### Patient management and outcomes

Patients were either directly administered to our stroke center or transferred from other primary stroke centers. Indication for EVT is made interdisciplinary by the responsible neurologist and neuro-interventionalist and is offered 24/7. Experienced interventionalists chose thrombectomy technique and stenting material based on each individual patient case. EVT was performed under general anesthesia in all patients according to our standard protocol.^19^ At the end of the EVT, the interventionalist defined the TICI score for each patient by interpreting the final angiographic series. After termination of EVT all patients were transferred—intubated and mechanically ventilated—to our neurointensive care unit. Analgosedation post-interventionally was performed according to institutional protocol and terminated as soon as possible with consecutive fast extubation. Monitoring and stroke work-up was performed to standard institutional protocol and in accordance with current guidelines. Certified stroke nurses or neurologists obtained NIHSS scores at least every 6 h daily for at least 72 h in all patients.

All patients were screened for the following clinical complications: hemorrhagic transformation, pneumonia, symptomatic bradycardia, duration of intubation and length of ICU stay. For outcome measurements mRS was recorded at time of dismissal by institutional certified neurologists and after 3 months either during follow-up appointments at our institution or in a telephone interview, carried out by trained neurologist.

### Primary outcomes

Primary outcome was the rate of favorable neurological outcome at 3 months comparing interventional and control group. To define favorable neurological outcome we used a dichotomized representation of the mRS, with a score of 0–2 representing a favorable outcome and a score of 3–6 representing poor neurological outcome.

### Secondary outcomes

For secondary outcomes, mean mRS was compared as well as shift analysis of mRS distribution at 3 months. In addition, mean body temperature at end of cooling phase 6 h post-intervention was compared between patients with mRS 0–2 vs 3–6. For safety analysis, mortality at 3 months was compared between groups.

### Statistics

Patient characteristics are expressed as mean and interquartile range (IQR) for ordinal variables and relative frequencies for nominal variables. Variables were tested for normal distribution. Groups were matched for age, sex, NIHSS at hospital arrival, ASPECTS on primary cerebral computed tomography scan (CCT), as well as TICI score with defined maximum range of deviation using nearest neighbor matching. For primary as well as shift analysis in secondary outcome we used the logistic regression analysis. Mean values for secondary outcome analysis (mRS, mean body temperature at 6 h analysis) as well as safety outcomes and patient characteristics were compared using Mann–Whitney U-test and t-test depending on normal distribution. Statistics were performed using the IBM SPSS statistics software, V.28.0.1.0.

## Results

### Patient and EVT characteristics

Twenty-two patients participated in the COTTIS-1 trial and were included in the interventional group of this matched-pair analysis. Consecutively, 44 matched control patients were included in the control group (Table 1). According to matching parameters median age (77 vs 76 years) and gender distribution (55% male vs 55% male), median NIHSS at admission (15 vs 15), median ASPECTS at admission (9 vs 9) as well as distribution of occlusion site and distribution TICI score were equally matched between the groups. In addition, rate of pre-interventional thrombolysis, need for proximal internal carotid artery stenting and time windows as last-seen-normal (LSN)-to-groin, door-to-groin, LSN-to-recanalization and door-to-recanalization times did not differ between groups (Table 2).

**Table 1:** Demographic data

Patient characteristics (*n* = 66)	Control group, *n* = 44	Interventional group, *n* = 22
Variable		
Age, years, median (range)	76 (70–84)	77 (70–83)
Gender, male, *n* (%)	24 (55)	12 (55)
Patients with risk factors, *n* (%)		
Hypertension	33 (75)	15 (68)
Coronary heart disease	4 (9)	7 (32)
Diabetes mellitus	8 (18)	5 (23)
Hyperlipidemia	13 (30)	8 (36)
Current smoker	9 (21)	4 (18)
Prior stroke	7 (16)	3 (14)
Reason for stroke, *n* (%)		
Atrial fibrillation	26 (59)	11 (50)
Proximal ICA stenosis	10 (23)	4 (18)
Unknown	8 (18)	5 (23)
Premedication, *n* (%)		
Aspirin	8 (18)	5 (23)
Oral anti-coagluation	9 (21)	6 (27)
Admission from rural hospital, *n* (%)	24 (55)	9 (41)
Admission year, median (range)	2022 (2021, 2022)	2018 (2014, 2021)

Table lists clinical data on patient history.

**Table 2:** Stroke characteristics

Variable	Control group, *n* = 44	Interventional group, *n* = 22
Left hemisphere, *n* (%)	20 (46)	11 (55)
Occlusion site of vessel, *n* (%)		
First segment of MCA (M1)	23 (52)	12 (55)
Second segment of MCA (M2)	5 (11)	3 (14)
Tandem occlusion	6 (14)	4(18)
Distal ICA + M1	10 (23)	3 (14)
ASPECTS at admission, median (range)	9 (8.25–10)	9 (8.25–10)
rtPA given, *n* (%)	24 (55)	14 (64)
NIHSS at admission, median (IQR)	15 (12–18)	15 (12.5–19.75)
Reperfusion, *n* (%)		
TICI 0–1	0	2 (9)
TICI 2a	3 (7)	0
TICI 2b	12 (27)	11 (50)
TICI 2c/3	29 (66)	9 (41)
Times (min) from x to x, median (IQR)		
LSN-to-recanalization	387,51 (226–434)	355 (258–645)
LSN-to-groin	328.61 (149–342)	309 (228–580)
Door-to-groin	78.88 (58–79)	65.36 (40–82)
Door-to-recanalization	142.51 (101–180)	123 (93–140)

Table lists stroke characteristics (*n* = 66) by groups.

Abbreviations: MCA = middle cerebral artery; rtPA = rekombinant tissue plasminogen activator.

### Temperature course

**Figure 1:**
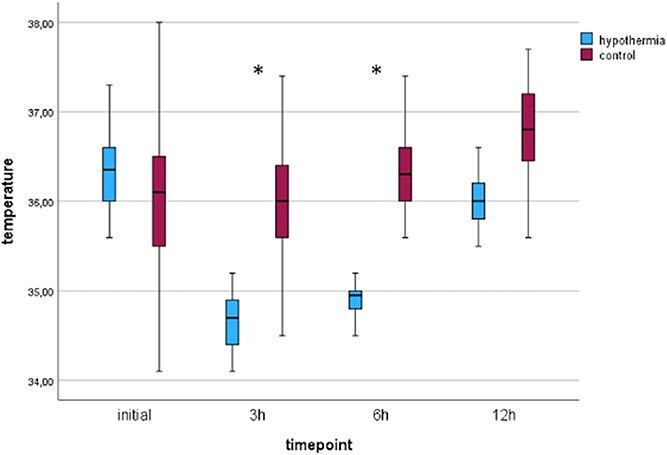
Temperature profiles. Figure displays temperature profiles of interventional (COTTIS) and control group during the first 12 h after admission. ^*^p < 0.001.

Temperature profiles are shown in Figure 1. At admission, the median temperature did not differ between groups. After induction of hypothermia by transnasal cooling body temperature reached target temperature of 35°C in 30 min (range 15–78 min; cooling rate 2.6°C/h) in the interventional group. During this phase there are no recorded temperatures in the control group but first measured body temperature at 3 h post-EVT on the ICU differed significantly with a median temperature of 36°C in the control group and 34.7°C in the interventional group (*p* < 0.001), as well as at 6 h post-EVT (median temperature 34.95°C vs 36.3°C (*p* < 0.001)).

### Primary outcomes

**Figure 2:**
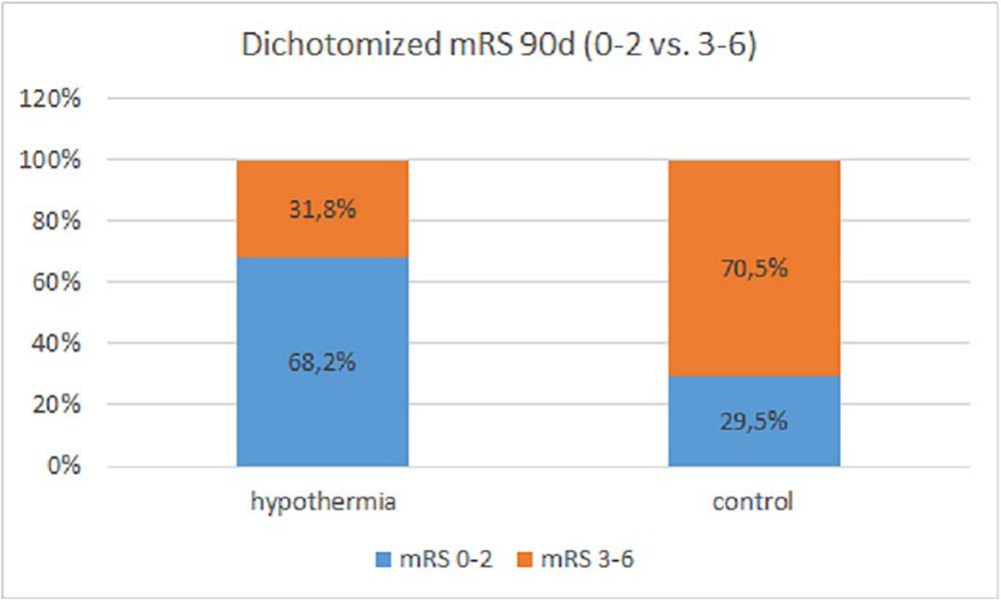
Dichotomized mRS at 90 days. Figure displays primary endpoint with dichotomized mRS 0–2 versus 3–6 at 90 days (*p* = 0.004).

A favorable neurological outcome defined as mRS 0–2 was reached significantly more often in patients in the interventional group compared with patients in the control group (15/22 vs 13/44) with an unadjusted odds ratio (OR) of 5.1 [95% confidence interval (95% CI) 1.69; 15.38] (*p* = 0.004) (Figure 2).

### Secondary outcomes

**Figure 3:**
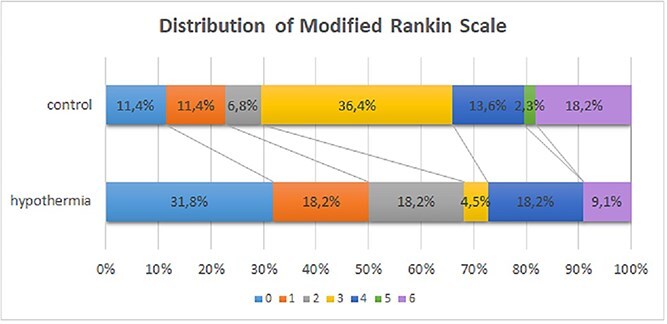
Distribution of mRS at 90 days. Figure displays secondary endpoint with distribution of mRS at 90 days and shift of mRS categories (*p* = 0.004).

Secondary outcome analysis included a mRS shift-analysis showing that patients in the interventional group had a significant shift towards better outcomes compared with patients in the control group (Figure 3): in therapeutic hypothermia the probability of attaining a higher score on the mRS was reduced compared with normothermic controls with an unadjusted OR of 0.225 (95% CI 0.081, 0.628) (*p* = 0.004).

**Figure 4:**
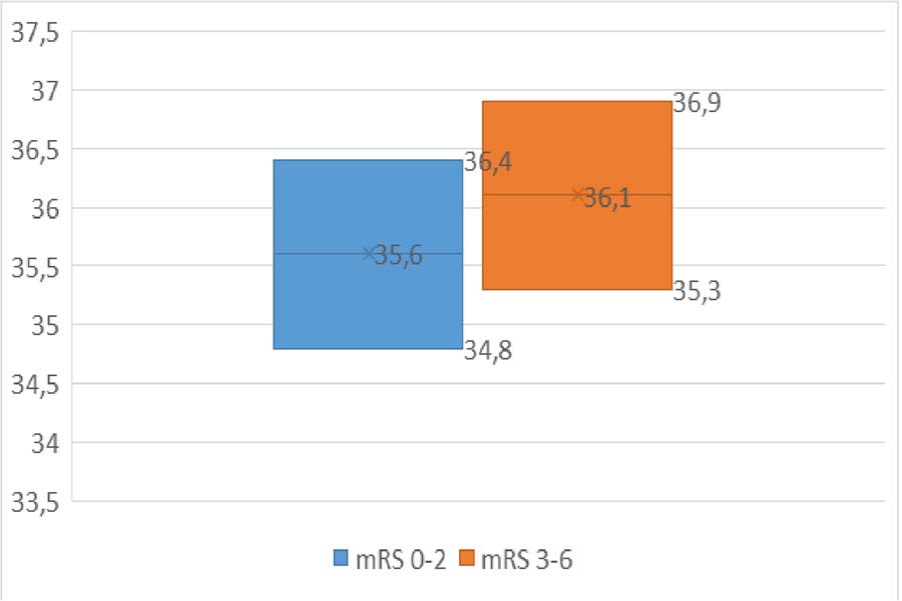
Outcome associated body temperature at 6 h. Figure displays body temperature at 6 h post-admission in favorable (mRS 0–2) vs unfavorable (mRS 3–6) 90-day outcomes.

Mean mRS values at 3 months were significantly lower in the interventional group (1.95 vs 3.09) (*p* = 0.025). Comparing median body temperature at 6 h of patients with favorable neurological outcome to patients with unfavorable outcome, body temperature was significantly lower in favorable outcome [35.6°C (34.8; 36.4) vs 36.1°C (35.3; 36.9)] (*p* = 0.025) (Figure 4).

### Safety outcomes

Mortality at 3 months was lower in the interventional group (9.1% vs 18.2%) but did not reach statistical significance. Other safety outcomes as symptomatic intracranial hemorrhage, pneumonia, hemodynamic instability and cardiac arrhythmia or renal function disturbance did not differ between groups. In addition, device-related complications within the interventional group as smell and tasting disturbance or nose bleeding did not occur (see COTTIS-1^17^).

## Discussion

The introduction of mechanical thrombectomy into the standard therapeutic regimen for acute LVO has yielded substantial improvements in patient outcomes. A notable 46% of patients have demonstrated a favorable clinical outcome, defined as mRS scores of 0–2, within 90 days.^1^ Despite this highly effective therapy, a considerable proportion of patients still has an unfavorable outcome, primarily attributable to inadequate collateral circulation and the progression of infarct core size until orthograde blood supply is restored through recanalization of the occluded vessel. In order to extend the intra-interventional time window until final recanalization and to reduce reperfusion injury, the implementation of additional neuroprotective strategies is necessary. We used intra-ischemic rapid induction of mild hypothermia via transnasal cooling followed by 6 h hypothermia after recanalization by surface cooling as a potent and ubiquitous neuroprotectant to improve neurological outcome.

By comparing patients with peri- and post-interventional hypothermia to matched controls without cooling we were able to show that the use of mild hypothermia is significantly more likely to result in a good clinical outcome. Our findings align with the results of mainly preclinical studies that have highlighted the neuroprotective effect of hypothermia in the treatment of acute stroke. This is attributable to the multifaceted underlying mechanisms of hypothermia, which address the pathophysiological processes associated with acute stroke.^16,20–22^ The efficacy of this effect appears particularly promising in the context of mechanical thrombectomy, as recanalization is achieved in a large proportion of patients and accordingly others describe a positive correlation between the use of hypothermia and clinical recovery after thrombectomy.^23^

Earlier clinical trials on the implementation of therapeutic hypothermia in AIS encountered several problems and lead to negative results as a consequence. The hypothermia protocol that was implemented was designed to address these issues and has the potential to result in a substantial improvement in clinical outcomes. Initially, it appears imperative to identify suitable patient cohorts, which are likely to profit from the use of hypothermia. Studies identified reperfusion as a precondition for hypothermia-induced neuroprotection.^24^ Given the considerable tissue at risk in patients with LVO and the high recanalization rates observed in LVO and EVT, this patient population resembles experimental trials on hypothermia in AIS which proved to be highly beneficial.^25^ Secondly, a number of clinical studies were terminated due to inadequate participant enrolment, inadequate understanding of therapeutic hypothermia or simply due to unattainability of the set temperature targets^26^ or massive delay of induction. Hence induction and maintenance of hypothermia in stroke patients must be fast, simple and minimally invasive. By using intranasal cooling in the interventional group all prerequisites were met as a basis for fast induction of hypothermia with a cooling rate of 2.6°C/h. Furthermore clinical trials observed sometimes severe side effects of hypothermia especially when inducing hypothermia in awake patients or prolonged duration of hypothermia, which may reduce the protective effects of hypothermia.^26^ Side effects increase with the duration and depth of hypothermia and are rarely seen during short lasting hypothermic treatment in intubated patients after cardiac arrest.^15,27^ Therefore, within the interventional cohort, hypothermia was constrained to a maximum of 35°C for a duration of 6 h following recanalization to minimize these side effects and establish neuroprotectant covering of both the intra-ischemic and early reperfusion phase. With this approach we were able to show that peri-interventional mild hypothermia has the potential to minimize neuronal damage, promote functional recovery and significantly improve outcome.

After recanalization in AIS, patients are at risk of cerebral reperfusion injury.^28,29^ Pathophysiological processes related to reperfusion injury are influenced on different levels by hypothermia which has been described and discussed in reperfusion following stroke as well as global cerebral ischemia (posthypoxic cerebral damage), e.g., stroke and hypoxic damage.^30^ Thus, extending hypothermia for 6 h post-recanalization may have exerted a beneficial effect on the outcomes observed in the interventional cohort within the present study.

The discrepancy in outcomes observed in this study, in contrast to those reported in studies on accidental hypothermia during thrombectomy which demonstrated a deleterious effect of hypothermia on outcomes, may be attributed to the extension of hypothermia treatment into the post-interventional phase. In those studies, patients were rapidly warmed after thrombectomy, potentially during the critical reperfusion phase.^31,32^ This is a significant difference from our study, which found improved outcomes in the group with peri-interventional hypothermia. Another difference from retrospective analyses of accidental hypothermia during thrombectomy is that hypothermia was rapidly induced in our study. The target temperature of 35°C was reached within 30 min. Conversely, the occurrence of hypothermic temperatures during thrombectomy in accidental hypothermia remains unclear.

An analysis of the temperatures across the entire study cohort revealed a significantly lower body temperature in patients with favorable outcome. Others^25,33^ have shown similar correlations in their analysis, highlighting the need to define the optimal temperature targets for hypothermia in future studies as they conclude that targeted temperature management during thrombectomy may reduce neuronal damage and improve recovery rates. In the present study the interventional group was maintained at a target temperature of 35°C which has been demonstrated to exhibit a significant neuroprotective effect in several studies underlining the effect that was seen in our study despite a mean temperature difference of only 1°C between groups. Side effects associated with mild hypothermia on the other hand remain within a low range with a target temperature of 35°C, and increase significantly with lower target temperatures.^26,34^ Additionally, the rewarming phase is significantly shorter with target temperatures of 35°C compared with 34°C or even lower, which translates into shorter intubation times which are necessary in hypothermic treatment. This is meaningful for stroke patients, since prolonged intubation times post-mechanical thrombectomy are associated with worse outcomes.^35^ Therefore, by establishing a target temperature of 35°C, rewarming times were minimized to ensure expeditious extubation.

In the present study, the use of a target temperature of 35°C combines the positive effects of hypothermia, such as neuroprotection and a positive influence on reperfusion injury, with the lowest possible hypothermia-associated side effects and the shortest possible intubation phase following a brief rewarming period. This is reflected in the safety data from the COTTIS-1 study and the lack of difference in complication rates between treatment groups. Furthermore, the present study exhibited a lower mortality rate, though the difference between the hypothermic intervention group and the control group was not significant. These results align with the findings of others^36^ which highlight the safety of mild hypothermia in acute stroke therapy. The fact that no significant differences in complication rates were found suggests that the use of mild hypothermia can be considered safe. However, it is important to consider the long-term effects and potential risks of this therapy as has been shown by others.^26,34^

## Limitations

There are some limitations to this matched-analysis study: we did not match our control group for time-of-onset to treatment for stroke. Given that this time is an important predictor in outcome and a potential confounder in our results, this might limit our findings. Nevertheless, there were no differences in treatment times despite non-significant deviations. In particular, the important time intervals to recanalization (LSN-to-recanalization, door-to-recanalization, groin-to-recanalization) did not differ between groups. Additionally, as we matched all our patients for initial ASPECTS, we created similar conditions for the severity of stroke in both groups, which might compensate this non-significant difference in treatment windows*.* As the data sampling in the control group was retrospective there are missing data regarding patients’ temperature upon admission for four patients (9%), which limits comparison between temperature profiles between the groups. However, none of these patients showed spontaneous hypothermia with arrival at the ICU, indicating that these patients had physiological temperature profiles during intervention. Another relevant confounder may be the differences in the treatment period. Patients in the hypothermic group were treated mainly in 2022 whilst patients in the control group were treated at median treatment year of 2018, ranging from 2014 to 2021. Since EVT has improved considerably over time, in addition to improvements in standard stroke care, this might have influenced outcome differences between groups. Also, the influence on outcome of a carefully selected trial cohort such as the hypothermia group compared with the unselected real world data in the control group needs to be considered. Finally, despite a small sample size the effect size is surprisingly high, which needs critical review, and selection bias may be considered. In the hypothermic group selection bias was minimized by consecutive inclusion. To rule out selection bias for the control group we matched for multiple confounders of stroke outcome in EVT using nearest neighbor matching and additionally performed 2:1 matching to increase the validity of the analysis. Nevertheless, the evidence derived from our study is insufficient for direct application to clinical practice.

## Conclusion

Despite these limitations, peri-interventional mild hypothermia in AIS seems to have a beneficial effect on outcome, with higher rates of good clinical outcomes compared with non-hypothermic patients undergoing mechanical thrombectomy. Thus, randomized controlled trials (COTTIS-2) are needed to underline these findings and demonstrate the efficacy of periinterventional mild hypothermia on the neurological outcome in AIS and mechanical thrombectomy.
